# Neural Systems with Numerically Matched Input-Output Statistic: Isotonic Bivariate Statistical Modeling

**DOI:** 10.1155/2007/71859

**Published:** 2007-07-12

**Authors:** Simone Fiori

**Affiliations:** Dipartimento di Elettronica, Intelligenza Artificiale e Telecomunicazioni, Università Politecnica delle Marche, Via Brecce Bianche, 60131 Ancona, Italy

## Abstract

Bivariate statistical modeling from incomplete data is a useful statistical tool that allows to discover the model underlying two data sets when the data in the two sets do not correspond in size nor in ordering. Such situation may occur when the sizes of the two data sets do not match (i.e., there are “holes” in the data) or when the data sets have been acquired independently. Also, statistical modeling is useful when the amount of available data is enough to show relevant statistical features of the phenomenon underlying the data. We propose to tackle the problem of statistical modeling via a neural (nonlinear) system that is able to match its input-output statistic to the statistic of the available data sets. A key point of the new implementation proposed here is that it is based on look-up-table (LUT) neural systems, which guarantee a computationally advantageous way of implementing neural systems. A number of numerical experiments, performed on both synthetic and real-world data sets, illustrate the features of the proposed modeling procedure.

## 1. INTRODUCTION

Most complex, real-world systems (possibly having stochastic elements) and phenomena cannot be accurately described by a mathematical model to be evaluated analytically. In this case, statistical modeling provides a useful tool to build up a model of the phenomenon under observation. The classical theory of stochastic modeling bases on concepts such as Markov chains, renewal theory, and queuing theory [1, 2]. Known applications range from physics [3] to automatic speech recognition and language translation [4], from broadband communications systems [5] to magnetic hysteresis modeling [6] and economics [7].

Also, statistical modeling pays off in those applications where incomplete data only are available. The problem of incomplete data is a serious one in many fields of research. In general, data being incomplete means that specific observations are either lost or are not recorded exactly. Based on the definitions adopted in the insurance context (see, e.g., [8]) there are two main ways in which the data can be incomplete.

**Censored data**

Data are said to be censored when the number of observations that fall within a given set is known, but the specific values of the observations are unknown.

**Truncated data**

Data are said to be truncated when observations that fall within a given set are excluded.

While the censored-data definition implies that only the number of observations was recorded, the truncated-data definition implies that neither the number nor the values of such observations were recorded (e.g., in the operational risk field the second scenario is the most common one: the truncated data refer to the recorded observations all of which fall above a positive threshold of a specific amount, while the missing data identify the unrecorded observations falling below the known threshold). The latter are usually called non-randomly missing data, to distinguish them from the randomly missing data that may instead affect the observations that fall over the entire range of the data and can be caused, for example, by an inadequate data collection process.

Little and Rubin [9] defined three types of incomplete data (see also [10]).

**Missing completely at random (MCAR)**

This is the case when there are missing records in a data set but there is no chance to guess that such records are actually missing.

**Missing at random (MAR)**

Cases with incomplete data differ from cases with complete data, but the pattern of incomplete data is traceable or predictable from other variables in the data sets. In other words, the actual variables where data are missing are not the cause of the incomplete data: instead, the cause of the missing data is due to some external influences.

**Nonignorable**

The pattern of incomplete data is not random nor is it predictable from other variables in the data sets. In contrast to the MAR situation, where incomplete data can be traced on the basis of other measured variables, nonignorable missing data are only explainable by the very variables the values of which are missing.

In practice, it is difficult to meet the MCAR assumption, while the MAR assumption is more reasonable oftentimes. The more relevant and related predictors one can include in statistical models, the more likely it is that the MAR assumption will be met.

Missing-data imputation and handling is a rapidly evolving field counting many methods, each applicable in some specific circumstances. When choosing a missing data handling approach, one of the desired outcomes is maintaining (or approximating as closely as possible) the shape of the original data distribution. Some incomplete data handling methods are superior to others in maintaining distributions' shape. The following list covers some of the more widely recognized approaches to handling data sets with incomplete cases [9, 11, 12].

- Listwise or casewise data deletion: if a record has missing data for any variable used in a particular analysis, omit that entire record from the analysis.
- Pairwise data deletion: for bivariate modeling, compute statistics based upon the available pairwise data only.
- Mean substitution: substitute a variable's mean value computed from available cases to fill in missing data values on the remaining cases.
- Hot deck imputation: identify the most similar case to the case with a missing value and substitute the most similar case's value for the missing case's value.
- Expectation maximization (EM) approach: an iterative procedure that proceeds in two discrete steps. In the expectation step, compute the expected value of the complete data log likelihood. In the maximization step, replace the expected values for the missing data obtained from the expectation step and then maximize the likelihood function as if no data were missing to obtain new parameter estimates. Iterate through these two steps until convergence is achieved.
- Maximum likelihood (ML) approach: use the whole set of available data to generate maximum likelihood-based sufficient statistics. Usually, such statistics consist of variables covariance matrix and means.
- Multiple imputation: similar to the maximum likelihood method, except that multiple imputation generates data values suitable for filling in gaps in an existing database. Typically, several databases are created in this way and the investigator than analyzes these datasets using an appropriate statistical analysis method, treating these databases as if they were based on complete case data. The results from this analysis are then combined into a single summary finding.
- Regression methods: develop a regression equation based on complete case data for a given variable, treating it as the outcome and using all other relevant variables as predictors. Then, for cases where a variable is missing, plug the available data into the regression equation and substitute the equation's predicted value into the data set for subsequent use.

Comparisons of these methods, presented in [9, 11, 12], show that listwise, pairwise, and mean substitution missing data handling methods are inferior when compared with maximum likelihood or multiple imputation methods. Regression methods are somewhat better, but not as good as hot deck imputation or maximum likelihood approaches. The EM method falls somewhere amidst them, as it is generally superior to listwise, pairwise, and mean substitution approaches, but it lacks the uncertainty component contained in the maximum likelihood and multiple imputation methods.

In the present work, we proceed under the MAR assumption and consider bivariate *statistical modeling* with a method that differs from the ones recalled above (while partially resembling the regression method). Statistical modeling (also known “imputation” in the context of statistics) with incomplete data is a useful statistical tool that allows to infer the model underlying two data sets in the case that the data in the two sets correspond to each other in an unknown way. Such case may occur when the sizes of the two data sets do not match (i.e., there are “holes” in the data) or when the data have been acquired independently and a statistical analysis is to be launched in order to discover their dependency. Also, statistical modeling is useful when the amount of available data is sufficiently large to show relevant statistical features of the phenomenon underlying the data.

As an example of data sets *𝒟_x_* and *𝒟_y_* of different sizes, let us consider the following case. On a population of students, the set *𝒟_x_* represents the set of grades attributed by a teacher at the end of an exam and the set *𝒟_y_* represents the set of evaluation values *anonymously* attributed by the students to the teacher after class completion. About these data sets, the following observations can be made.

1. Not all the students might want to evaluate the teacher, so the size of data set *𝒟_y_* might differ from the population size.
2. For some special reason, the teacher might exclude some students from grading, therefore the size of data set *𝒟_x_* might differ from the population size.
3. Because of anonymity of student's evaluation, the scores in the data sets *𝒟_x_* and *𝒟_y_* come unpaired (namely, without any a priori link to each other).
4. It is tenable to deduct that the students which will receive the worst grades will also evaluate the teacher with low scores, therefore the model linking the values of the data sets *𝒟_x_* and *𝒟_y_* may be expected to exhibit a monotonically increasing shape.

The general problem of finding a monotonic regression model is known as “isotonic regression” in statistics [13].

**Figure 1:**
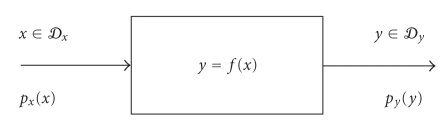
The neural system used for modeling along with relevant quantities of input/output data sets.

In the present paper, we consider tackling the problem of statistical modeling via a neural system that is able to match its own input-output statistic to the statistic of the available data sets. The neural system is shown in Figure 1, where *x* and *y* denote the variables whose analytic dependency is to be modeled, *𝒟_x_* and *𝒟_y_* denote the data sets that such variables belong to, while *p_x_*(⋅) and *p_y_*(⋅) denote the probability density functions that the variables *x* and *y* are drawn from. Neural systems exhibit nonlinear signal/data processing ability as well as adaptivity. In the present work, neural systems do not need training, so we retain the “neural” feature of nonlinear input/output transference only.

Unlike previous contributions such as [14, 15], which required deep theoretical grounds, in the present contribution, we focus on a conceptually simple algorithm and on efficient implementation issues. The key points of the proposed method may be briefly summarized as follows.

**(i) Modeling as statistic matching problem**

Statistical modeling is interpreted here as an input-output statistic matching problem for a nonlinear system (or neural network). Therefore, instead of considering the *x* ∈ *𝒟_x_* and *y* ∈ *𝒟_y_* variables as paired and to look for a nonlinear model that fits the best variables values, we consider the cumulative statistical information brought on by the data sets *𝒟_x_* and *𝒟_y_* in terms of their probability density functions. The neural system nonlinear transference function matches the probability distributions of the variables rather than the variables' values. This allows the proposed statistical modeling technique to cope with the modeling problem when the size of the two data sets does not match (case of missing data) or when the data sets were collected independently so the pairing relationship of the values within *x* ∈ *𝒟_x_* and *y* ∈ *𝒟_y_* is unknown (or was lost during the data-collection step).

**(ii) Simple and efficient numerical representation**

The quantity of interest as well as the learnt model are represented in terms of paired lists of numbers (namely, in terms of look-up tables, or LUTs). The LUTs provide an efficient way of representing and handling the variables appearing in the devised statistical modeling algorithm.

**(iii) Computational advantages**

As it will be clear later, advantages of the procedure devised on here are that: (1) it does not involve any computation except for LUT handling (which may be implemented by sorting/searching on lists of numbers) and few simple algebraic operations on numbers; (2) from the viewpoint of implementation on a “number cruncher,” the operations on the data sets *𝒟_x_* and *𝒟_y_* and may be performed in a parallel way until the very last step.

**(iv) Fundamental limitation**

As an inherent restriction of the method, the developed theory as well as its numerical implementation requires the modeled relationship to be bijective, namely, it requires the model to be monotonic (increasing or decreasing), while its shape is otherwise unrestricted. As the model *f*(⋅) is free of any shape constraint except for monotonicity, the related modeling procedure is free of bias effects inherently tied to other parametric modeling methods.

**(v) Interchangeability of pooled data and statistic**

As the individual data in the sets *𝒟_x_* and *𝒟_y_* are not used directly but only cumulative statistical functions obtained by pooling such data take part in the modeling procedure, if the data sets of the phenomena under investigations are not available but only their pooled cumulative statistical functions are available, the modeling process considered here may nevertheless be exploited.

The paper is organized as follows: Section 2 briefly reviews some relevant scientific literature related to the topic of the present manuscript. Section 3 discusses the modeling problem in details and presents the related analytic setting and its solution; the section then describes the required operations with look-up tables and then presents the numerical procedure of statistical modeling corresponding to the devised problem setting and analytic solution. Section 4 presents the results of numerical experiments obtained with synthetic data sets as well as real-world data sets. In particular, an experiment on synthetic data sets allows us to compare the modeling result obtained with the method proposed on here with the results obtainable with an algorithm for random number generation proposed in [16]. The experiments with real-world data sets allow us to gain some insights into the behavior of the neural-system-based statistical modeling method when coping with real-world data. In particular, statistical polypropylene matrix composite reinforced with natural fiber data results may be compared to the model presented in the previous work [17].

## 2. SHORT REVIEW OF STATISTICAL MODELING AND ITS APPLICATIONS

In this section, we present a short review of contributions addressing statistical modeling using pairwise interactions that share the same features of the approach presented in the present manuscript, namely information theoretical learning (ITL, [18]), as well as selected contributions that show various applications of statistical modeling. A topic that is also related to the content of the present paper is nonparametric kernel regression, although such method looks quite different in terms of implementation being based on matrix operators. We also refer to interesting recently published surveys for the sake of readers' convenience.

Some selected contributions drawn from the scientific literature are worthwhile considering to get a painting of current applications of statistical modeling.

**Data filling from incomplete oceanographic data sets [19]**

This paper presents a self-consistent method to infer missing data from oceanographic data series. The method presented in [19] allows to detect the number of statistically significant empirical orthogonal functions by a cross-validation procedure for a complete or incomplete data set as well as the noise level and interpolation error. Orthogonal functions may be gotten by, for example, singular value decomposition of covariance matrices. Since for the proposed filling and analysis method there is no need for a priori information about the error covariance structure, the method is self consistent and parameter free.

**Imputation strategies for blood pressure data [20]**

Underlying or untreated blood pressure is often an outcome of interest but is unobservable when study participants are on anti-hypertensive medications. Untreated levels are not missing at random but would be higher among subjects on medication. In such cases, standard methods of analysis may lead to bias. Blood pressure data obtained at the private physician's office (“out-of-study blood pressure data”) at the time of prescription of anti-hypertensive medications may be used to adjust for the potential bias. In particular, in [20], such data were used to estimate the conditional expectation and variance of the unobserved nonmedicated study blood pressure data. For those with no physician data, imputation from bootstrap samples of out-of-study blood pressure data was used: an iterative method based on the EM algorithm was employed to estimate the unknown study parameters in a random-effects model. This model was compared in [20] to an alternative model for the out-of-study blood pressure data based on a theoretical truncated normal distribution. Differences between methods were observed in the decline in blood pressure over time in the reference group: estimated intervention effects tended to be slightly larger using the imputation methods.

**On the use of missing-data methods in psychosomatic medicine [21]**

This paper summarizes recent methodological advances related to missing data and provides an overview of maximum likelihood estimation and multiple imputation. The paper carries on an overview of missing data theory: brief descriptions of traditional missing data techniques are given and maximum likelihood estimation as well as multiple imputation methods are outlined with a great deal of details. Special attention is paid within the manuscript to an analytic strategy that allows incorporating auxiliary variables into the analytic model. The paper [21] concludes with an illustrative analysis using an artificial quality-of-life data set.

**Estimation of social practices diffusion processes from incomplete data [22]**

Event-history analysis of the diffusion of practices in a social system can show how actors are influenced by each other as well as by their own characteristics. The presumption that complete data on the entire population are essential to draw valid inferences about diffusion processes has been a major limitation in empirical analysis and has precluded diffusion studies in large populations. The authors examine the impacts of several forms of incomplete data on estimation of the heterogeneous diffusion model proposed by Strang and Tuma [23]. Left censoring causes bias, but right censoring leads to no noteworthy problems. Extensive time aggregation biases estimates of intrinsic propensities but not cross-case influences. Importantly, random sampling can yield good results on diffusion processes if there are supplementary data on influential cases outside the sample. Paper [22] concludes that the capability of obtaining good estimates from sampled diffusion histories should help to advance research on diffusion.

**Handling missing data in educational research [24]**

Recently, missing data analysis techniques have received considerable attention in the methodological literature, where multiple imputation and maximum likelihood estimation are recommended. The article [24] provides an overview of missing-data theory, maximum likelihood estimation, and multiple imputation. Also, the paper carries on a methodological review of missing-data reporting practices drawn from a number of applied research journals. Reference [24] then provides a demonstration of multiple imputation and maximum likelihood estimation using the “longitudinal study of American youth data.”^1^ The results indicate that explicit discussions of missing data increased substantially between 1999 and 2003, but the use of maximum likelihood estimation or multiple imputation was rare: the studies relied almost exclusively on listwise and pairwise deletion.

**Analysis of incomplete climate data [25]**

The expectation maximization (EM) algorithm, as an iterative regression method from incomplete datasets and for the imputation of missing values, is taken in [25] as the starting point for the development of a regularized EM algorithm. In contrast to the conventional EM algorithm, the regularized EM algorithm is applicable to sets of climate data, in which the number of variables typically exceeds the sample size. The regularized EM algorithm is based on iterated analysis of linear regressions of variables with missing values on variables with available values, with regression coefficients estimated by ridge regression. Ridge regression is a regularized regression method in which a continuous regularization parameter controls the filtering of the noise in the data. The regularization parameter is determined by generalized cross-validation, such as to minimize, approximately, the expected mean-squared error of the imputed values. The regularized EM algorithm can estimate, and exploit for the imputation of missing values, both synchronic and diachronic covariance matrices, which may contain information on spatial, stationary temporal covariability or cyclostationary temporal covariability. Results of experiments reported in [25] demonstrate that the algorithm is applicable to typical sets of climate data and that it leads to more accurate estimates of the missing values than a conventional noniterative imputation technique.

A fertile research field involving probability modeling from incomplete data is the one of classification with labeled, unlabeled, and labeled with constraints. A recent view of this topic emerge, for example, from [26–28], which deal with discriminative learning in belief-net classifiers, classification by pairwise coupling and clustering by boosted mixture models.

Some contributions drawn from the scientific literature related to pairwise interactions (namely, on the subject of information theoretical learning) are worthwhile considering.

**Learning from examples with information theoretic criteria [29]**

This paper discusses a framework for adaptive systems learning based on information theoretic criteria. A novel algorithm based on Renyi's quadratic entropy is used to train, directly from a data set, adaptive systems for entropy maximization or minimization. An analogy between the computation and an information potential measuring the interactions among the data samples (pairwise interaction) is explained. The newly proposed criterion is tested in blind source separation (unsupervised learning) and in feature extraction for classification (supervised learning).

**Information potential for adaptive system training [30]**

This paper shows that using entropy criterion for learning provokes the minimization of the average information content of the error signal rather than merely trying to minimize its energy. The authors of [30] propose a generalization of the error entropy criterion that enables the use of any order of Renyi's entropy and any suitable kernel function in density estimation. The equivalence between global optimization by convolution smoothing and the convolution by the kernel in Parzen windowing is also discussed. Simulations presented in [30] concern time-series prediction and classification.

**Joint statistical models for audio-visual fusion [31]**

Audio and visual signals arriving from a common source may be detected using a signal-level fusion technique. By comparing the mutual information between different pairs of signals, it is possible to identify which person is speaking a given utterance and to get rid of errant motion. To this aim, a probabilistic multimodal generation model may be introduced and used to derive an information-theoretic measure of cross-modal correspondence. Reference [31] makes use of nonparametric statistical density modeling techniques to characterize the mutual information between signals from different domains.

**Search algorithms for information-theoretic learning [32]**

Research papers have proposed various ITL criteria based on Renyi's quadratic entropy with nonparametric kernel-based density estimation as alternative performance metrics for both supervised and unsupervised adaptive system training. The drawback of these information-based metrics is the increased computational complexity. The authors of [32] examine known parameter-search algorithms, like gradient-descent methods, conjugate gradient, approaches and the Levenberg-Marquardt algorithm, and propose modifications to allow training of systems with these ITL criteria.

Recent reviews, surveys, and essays drawn from the scientific literature about statistical modeling are worthwhile considering here.

**Statistical modeling in economics and finance [33]**

This book provides a broad and rich cross-section of contemporary approaches to statistical modeling in finance and economics and it is decision-making oriented. Covered topics range from common tools to solutions of sophisticated system problems and applications. In particular, a part of [33] is devoted to the allocation of funds and risk management. Another part explains modeling aspects of multistage stochastic programming, including a survey of parametric, multiobjective, and dynamic programming.

**Applied nonparametric regression [34]**

This book brings together techniques for regression curve smoothing involving more than one variable. It focuses on the applications and practical problems of two central aspects of curve smoothing: the choice of smoothing parameters and the construction of confidence bounds. The methods covered in [34] are shown to possess numerous applications in many areas using statistical analysis. Examples are drawn from economics, medicine, and engineering.

**Review of missing data handling [35]**

The authors of this manuscript frame the missing-data problem, review methods, offer advice, and raise issues that remain unresolved. They strive to clear up common misunderstandings regarding the MAR concept and summarize the evidence against older procedures. Also, the authors of [35] present two general approaches that come highly recommended, namely, maximum likelihood and Bayesian multiple imputation. Recent developments are discussed, including some new techniques to deal with missing data that are not MAR.

**Review of randomized controlled trials in medical journals [36]**

Randomized controlled trials in medicine almost always have some individuals with missing outcomes. Inadequate handling of these missing data in the analysis can cause substantial bias in the estimates of the effects of a treatment. The paper examines how missing outcome data are handled in randomized controlled trials in major medical journal in order to assess whether adequate steps have been taken to reduce nonresponse bias and to identify ways to improve procedures for missing data. The authors of [36] focused on trial designs, how missing outcome data were described, and the statistical methods used to deal with the missing outcome data.

A review of kernels theory/usage in adaptive systems may be found in [37]. We conclude the present review section with the list of some relevant contributions that discuss the general question of parametric/nonparametric modeling and of kernel-based regression techniques.

**Kernel regression under monotonicity constraints [38]**

The authors suggest a method for monotonizing general kernel-type estimators, for example, local linear estimators and Nadaraya-Watson estimators (see, e.g., [39]). The proposed approach is shown to produce smooth estimates. Implementation involves only an off-the-shelf programming routine. The method is based on maximizing fidelity to the conventional empirical approach, subject to monotonicity.

**Comparing parametric and nonparametric fits [40]**

In general, there are visible differences between a parametric and a nonparametric regression estimate. It is therefore of interest to compare these in order to decide whether the parametric model could be justified. An asymptotic quantification is the distribution of the integrated squared difference between these curves. Authors of [40] show that the standard way of bootstrapping this statistic fails. They use and analyze a different form of bootstrapping for this task, termed “wild bootstrap.”

**Hypothesis tests for statistical dependency [41]**

Determining the structure of dependencies among a set of variables is a common task in many signal and image processing applications, including multitarget tracking and computer vision. In this paper, authors present an information-theoretic, machine learning approach to problems of this type. Reference [41] casts this problem as a hypothesis test between factorizations of variables into mutually independent subsets and shows that the likelihood ratio can be written as sums of two sets of Kullback-Leibler divergence terms: the first set captures the structure of the statistical dependencies within each hypothesis, whereas the second set measures the details of model differences between hypotheses. The authors consider the case when the signal prior models are unknown, so that the distributions of interest must be estimated directly from data, showing that the second set of terms is (asymptotically) negligible and quantifying the loss in hypothesis separability when the models are completely unknown. They demonstrate the utility of nonparametric estimation methods, providing a framework for determining and distinguishing between dependency structures in highly uncertain environments. Additionally, the authors develop a machine learning approach for estimating lower bounds on Kullback-Leibler divergence and mutual information from samples of high-dimensional random variables for which direct density estimation is infeasible. The authors of [41] present empirical results in the context of three prototypical applications: association of signals generated by sources possessing harmonic behavior, scene correspondence using video imagery, and detection of coherent behavior among sets of moving objects.

**Chance probability functions in shape retrieval and classification [42]**

Several example-based systems for shape retrieval and shape classification directly match input shapes to stored shapes, without using class membership information to perform the matching. Reference [42] proposes a method for improving the accuracy of this type of systems. First, the system learns a set of chance probability functions, which represent the probabilities of obtaining a query shape with particular distances from each training example by chance. The learnt functions are used at runtime to rapidly estimate the chance probabilities of the observed distances between the actual query shape and the database shapes. These estimated probabilities are then used as a dissimilarity measure for shape retrieval and/or nearest-neighbor classification. The chance probability functions learning method is parameter free. Experimental evaluation demonstrates that chance probabilities yield higher accuracy than Euclidean distances, that the learnt chance probability functions support fast matching, and that the chance-probability-functions-based system outperforms prior systems on a standard benchmark test of retrieval accuracy.

## 3. STATISTICAL MODELING BY INPUT-OUTPUT STATISTIC MATCHING

The present paper completes the research program initiated with the probability density function estimation method proposed in [14, 43] and the neural nonlinear transference learning method for input-output density matching discussed in [16], which improves a previous algorithm recently published in [15].

In particular, the papers on probability density function learning were devoted to the development of methods that are capable of providing analytic (i.e., smooth) approximations of probability density functions of data sets on the basis of the available samples. Also, the nonlinear transference learning methods were developed in order to numerically estimate nonlinear models that transform a given probability density function into another known probability density function, for input-output density function matching, under the assumptions that the involved density functions are known in analytic form. Therefore, the latter class of methods is mixed analytic/numerical.

In statistical modeling, however, the input/output probability density functions are unknown and need to be estimated on the basis of the available input/output data sets. As the model-inference procedure is intrinsically numerical, it is appropriate to employ a discrete probability density function estimation algorithm.

Section 3.1 below reviews the modeling problem in an analytic fashion and sets the framework for the development of the actual numerical learning procedures, as discussed in the subsequent sections. The last section discusses the computational complexity issue.

### 3.1. Themodeling problem: analytic setting and solution

A well-known effect of nonlinear neural systems is to warp the statistical distribution of its input variable. In particular, we suppose that the system under consideration has a nonlinear structure described by the transference *y* = *f*(*x*), where *x* ∈ *𝒳* ⊆ ℝ denotes the input, having probability density function *p_x_*(⋅), and *y* ∈ *𝓎* ⊆ ℝ denotes the output signal, having probability density function *p_y_*(⋅).

In the hypothesis that the neural system transference is strictly monotonic, namely *f*′(*x*) > 0 or *f*′(*x*) < 0, for all *x* ∈ *𝒳*, the relationship between the input distribution, the output distribution, and the system transfer function is known to be [44]:

(1) $$p_{y}(y)=\pm \frac{p_{x}(x)}{f^{\prime }(x)}{|}_{x=f^{-1}(y)},x\in 𝒳,$$

where *f* ^−1^(⋅) denotes the inverse of function *f*(⋅) and the sign “+” arises when the nonlinear function *f*(⋅) is monotonically *increasing* while sign “−” arises when the function *f*(⋅) is monotonically *decreasing*.

Usually, (1) is interpreted as an analysis formula, which enables us to compute the output distribution when the input distribution and the system transference function are known. However, the cardinal equation (1) may also be interpreted as a formula that allows for designing the nonlinear system when the distribution *p_x_*(⋅) is known and it is desired that the system responds according to a desired distribution *p_y_*(⋅). In fact, (1) may be rewritten as the differential equation

(2) $$f^{\prime }(x)=\pm \frac{p_{x}(x)}{p_{y}(f(x))},\;x\in 𝒳,$$

in the unknown *f*(⋅). In order for the above equation to make sense, we require the function *p_y_*(⋅) to be nonzero in the domain of interest of the variable *y*. In general, solving (2) in the unknown *f*(⋅) implies solving a nonlinear differential equation, provided that a consistent boundary condition is specified.

Let us now define the cumulative distribution functions associated to the input-output probability density functions^2^:

(3) $$P_{x}(x)\overset{\text{def}}{=}\int_{-\infty }^{x}p_{x}(t)dt,\;\;\;P_{y}(y)\overset{\text{def}}{=}\int_{-\infty }^{y}p_{y}(t)dt.$$

In the case that the cumulative distribution functions associated to the input-output probability density functions are assumed to be known, the solution of the cardinal differential equation may be written in closed form as shown in [15]:

(4) $$f(x)=P_{y}^{-1}(c\pm P_{x}(x)),$$

where constant *c* depends on the boundary condition, symbol *P*_y_^−1^(⋅) denotes the inverse of the cumulative density function *P*_y_(⋅), and the sign in “±” is selected according to the desired sign for the derivative *f*′(⋅).

We consider right now the problem of boundary condition selection in the differential equation (2). The nonlinear function *f*(⋅) is sought for such that it maps the data distribution *p_x_*(⋅) into the distribution *p_y_*(⋅). Therefore, a sensible choice for the constant *c* in the model (4) is such that the model maps the center of mass of the *x* distribution into the mass center of the *y* distribution. If we denote by $\bar{x}$ and $\bar{y}$ the mean values of the *x* and *y* data sets, respectively, namely

(5) $$\overset{¯}{x}\overset{\text{def}}{=}\int_{-\infty }^{+\infty }t\cdot p_{x}(t)dt,\;\;\overset{¯}{y}\overset{\text{def}}{=}\int_{-\infty }^{+\infty }t\cdot p_{y}(t)dt,$$

then the mass-center-to-mass-center-map condition above writes $P_{y}(\bar{y})=c\pm P_{x}(\bar{x})$. As $P_{y}(\bar{y})=P_{x}(\bar{x})=1/2$, the above condition gives rise to the following solutions for the increasing/decreasing model type separately.

- *Monotonically increasing model*: the constant *c* takes on the value 0, therefore the solution (4) to (2) writes *f*(*x*) = *P_y_* ^−1^(*P*_x_(*x*)).
- *Monotonically decreasing model*: the constant *c* takes on the value 1, therefore the solution (4) to (2) writes *f*(*x*) = *P_y_* ^−1^(1 − *P*_x_(*x*)).

The problem of sign selection in (4) is solvable by reasoning on the nature of the physical phenomena underlying the involved data sets, as will be shown in the section devoted to numerical experiments.

The conclusion of the above problem setting and analytic solution is that, in order to develop a fully numerical method for statistical modeling for incomplete data, the following ingredients are of use: a suitable numerical estimation method for cumulative density functions and a suitable numerical format for function representation/handling (with particular emphasis on numerical function inversion).

### 3.2. Operations with look-up tables

**Figure 2:**
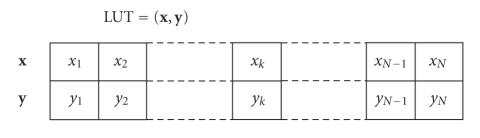
An *N*-size look-up table.

As already illustrated, for example, in [15, 16], look-up tables provide a completely suitable numerical representation for functions and probability-distributions-type quantities and related computations. A real-valued look-up table with *N* entries is basically a pair LUT = (**x**, **y**), where **x** ∈ ℝ^*N*^ and **y** ∈ ℝ^*N*^. The entries *x_k_* of **x** and *y_k_*, of **y**, with *k* ∈ {1,…, *N*} are paired and provide a pointwise description of an arbitrarily shaped function. An *N*-size look-up table is illustrated in Figure 2. It is worth noting that a look-up table is inherently undirected, namely, it represents a relationship between the *x* and *y* data rather than a dependency of the kind *y* = *y*(*x*) or *x* = *x*(*y*).

In order to handle the look-up tables for statistical modeling purpose, the following operations are of use.

**Cumulative sum**

On the basis of an *N*-point look-up table (**x**, **y**), a new look-up table may be constructed, whose *y*-entries contain the cumulative sum of the *y*-entries of the look-up table (**x**, **y**). Such operation is described by (**u**, **v**) = cumsum (**x**, **y**), where

(6) $$u=x,\;{\;v}_{h}=\overset{h}{\underset{k=1}{\sum }}y_{k},\;\;h\in \{1,\ldots \;,N\}.$$

The “cumsum” operator constructs a look-table (**u**, **v**) that has the same size of the argument look-table (**x**, **y**).

**Histogram computation**

In order to numerically approximate the probability distributions of one-dimensional numerical data sets, a histogram operator is of use. Let us denote by *𝒟* such data set (most likely represented by a number vector) and with *B* ≥ 2 the number of bins for frequency estimation. (Needless to say, data sets here are finite.) The histogram operation may be described by (**x**, **y**) = hist(*𝒟*, *B*). The constructed look-up table has *B* points and is built up as follows:

- *x_b_* equals the value of the *b*th bin center,
- *y_b_* equals the number of data points falling within the *b*th bin,
- *b* ∈ {1,…, *B*}.

The width of each bin is $\Delta_{x}\overset{\text{def}}{=}(\max\{𝒟\}-\min\{𝒟\})/B$. The bin centers are given by min{*𝒟*} + Δ_*x*_/2 + (*b* − 1)Δ_*x*_. The *b*th bin is the interval [min{*𝒟*} + (*b* − 1)Δ_*x*_ min{*𝒟*} + bΔ_*x*_), which is open on the right.

**Function inversion**

If a function is given a pointwise representation by the help of a look-up table (**x**, **y**), then its inverse function may be easily given a pointwise representation by the look-up table (**y**, **x**). Therefore, *function inversion in terms of look-up table representation does not require any computation at all*.

**Interpolation**

A limitation of LUT-based representation of functions is that the (*x*, *y*)-pairs for the represented relationship are known only on some points. Interpolation may be invoked to make computations with LUTs on other points in the domain. Interpolation may be performed in a variety of ways. In the present context, it is necessary to preserve the monotonicity of a function, therefore we refer here to linear interpolation only. Let us denote by *𝒟* the *x*-coordinate point set (most likely represented by a number vector) of size *D* ≥ 2, where the function represented by a look-up table (**x**, *y*) needs to be interpolated. The interpolation operation may be described by *ℓ* = interp(**x**, **y**, *𝒟*) and works as follows:

for every *d* ∈ {1,…, *D*}: *ℓ_d_* equals the interpolation of the *d*th *𝒟*-datum within
the LUT.

Of course, the built-up set *ℓ* is of size *D* and is to be considered an ordered set (which will likely be implemented as a vector, as well).

It is worth noting that the above definitions coincide as much as possible to the implementations of the corresponding operators provided by MATLAB, in order to make the computer implementation of the learning procedure as straightforward as possible. With the same spirit, in order to indicate that every entry of a vector is added with the same scalar, we use the usual vector-to-vector addition symbol (“+”).

### 3.3. Numerical procedure for statistical modeling

The model estimation procedure is based on a neural system, described by the input-output relationship *y* = *f*(*x*), where *x* ∈ ℝ denotes the input variable, *y* ∈ ℝ denotes the output variable, and *f*(⋅) denotes a neural transfer function.

The algorithm proposed in this section retraces the algorithm proposed in [16] for random-number generation. With respect to the algorithm proposed in [16], there are some important differences to be taken into account, which stem from the different use of the neural system made for random-number generation and for statistical modeling.

1. In random number generation, the input random variable is supposed to be drawn from a prototype distribution such as the uniform or Gaussian ones, which are simple and symmetric. The learning algorithm devised in [16] was written according to this assumption. In statistical modeling, however, both the input and output distributions may be arbitrarily involved, depending on the distribution of data within the *𝒟_x_* and *𝒟_y_* sets, therefore the learning algorithm should be generalized here accordingly.
2. The random number generation procedure devised in [16] consists of two separate stages: the first stage concerns neural system adaptation via a proper learning strategy; the second stage consists in using the learnt neural system as a generative model, in order to produce random numbers drawn from the desired statistic. In neural modeling, the second stage is apparently unnecessary, as the target of the method is to infer a model underlying the variables pair.
3. When a neural system is used for random number generation purpose, it is truly “directed,” in the sense that its input is supposed to be known (as it comes from an available prototype random number generator such as uniform or Gaussian) and its output can be computed only by passing the input through the learnt neural system. In statistical modeling, the variables of interest do not need to coincide necessarily to input and output variables, as we are seeking for a relationship among two variables to be “discovered.” This consideration is helpful as input and output variables may be swapped in order to obtain a model of the kind *x* = *f* ^−1^(*y*) *as well as* a model of the kind *y* = *f*(*x*), depending on which variable is to be considered as dependent from the other variable.

The look-up-table-based algorithm exploited here for numerical statistical modeling implements the closed-form design solution (4) by the help of the LUT-handling operators defined in Section 3.2

First, it is necessary to estimate the probability density functions as well as the cumulative distribution functions of data within *𝒟_x_* and *𝒟_y_* data sets. As already mentioned, in the interpretation of statistical modeling proposed in the present paper, the size *D_x_* of the data set *𝒟_x_* and the size *D_y_* of the data set *𝒟_y_* do not need to be equal. In order to perform a histogram-based estimation of the probability density function underlying the data via the “hist” operator, it is necessary to choose the numbers *B_x_* and *B_y_* of bins for the two data sets. We used the following rule of thumb to select the bins numbers:

(7) $$B_{x}\overset{\text{def}}{=}20\;\log_{10}D_{x},\;\;B_{y}\overset{\text{def}}{=}20\;\log_{10}D_{y},$$

where ⌈⋅⌉ rounds its argument to the nearest integer towards infinity. Such a choice endows the histogram-based estimation procedure with enough bins to estimate the probability density functions of the *x* and *y* data, while the number of bins keeps limited. The probability density functions of the *𝒟_x_* and *𝒟_y_* data sets may thus be numerically estimated by

(8) $$(x,p_{x})=\text{hist}({𝒟}_{x},B_{x}),\;\;(y,p_{y})=\text{hist}({𝒟}_{y},B_{y}).$$

It should be noted that the vectors **p**_*x*_ and **p**_*y*_ actually represent probability distributions up to scale factors, as they should be normalized by the total number of samples in each set and by the bin widths

(9) $$\begin{array}{c} {\text{Δ}}_{x}\overset{\text{def}}{=}\frac{\max\{{𝒟}_{x}\}-\min\{{𝒟}_{x}\}}{B_{x}},\\ {\text{Δ}}_{y}\overset{\text{def}}{=}\frac{\max\{{𝒟}_{y}\}-\min\{{𝒟}_{y}\}}{B_{y}}. \end{array}$$

The numerical cumulative distribution functions of the *𝒟_x_* and *𝒟_y_* data sets may now be estimated by numerical integration of the numerical probability density functions, which may be achieved by the help of the “cumsum” operator applied to look-up tables (**x**, **p**_*x*_) and (**y**, **p**_*y*_). It should be noted, however, that two adjustments are necessary at this point.

**(i) Lifting of *𝒟_y_*-set estimated distribution**

Some entries of the vector **p**_*y*_ may be zero (therefore some entries of its cumulative-sum vector may be equal to others). This situation violates the hypothesis that the probability density function of the *y* variable differs from zero in the interval of interest. Such occurrence should be fixed in a way that does not alter the information content of the vector **p**_*y*_ drastically. We propose, as a remedy, to add to every entry of the vector **p**_*y*_ a small quantity, namely 1/*D_y_*. Therefore, the normalized numerical probability density function to be integrated is

(10) $${\hat{p}}_{y}\overset{\text{def}}{=}(p_{y}+\frac{1}{D_{y}})\frac{D_{y}}{B_{y}+D_{y}^{2}}=\frac{D_{y}p_{y}+1}{B_{y}+D_{y}^{2}}(\approx \frac{p_{y}}{D_{y}}).$$

The entries of the vector **p**_*x*_ do not need any value-shifting, so the corresponding normalized numerical probability density function to be integrated is

(11) $${\hat{p}}_{x}\overset{\text{def}}{=}\frac{p_{x}}{D_{x}}.$$

It is easy to verify that the entries of vectors ${\hat{p}}_{x}$ and ${\hat{p}}_{y}$ sum up to 1.

**(ii) Shifting of cumulative distribution functions and bin centers**

The “cumsum” operator provides a cumulative-sum vector whose first entry coincides to the first entry of the summed-up look-up table, while the numerical cumulative distribution function's first entry should equal zero. Also, the “hist” operator provides the bin-centers coordinate, while for numerical function representation purposes, the boundary values of the bins are more easily profitable. Because of these reasons, we first define the look-up tables:

(12) $$(x,P_{x})=\text{cumsum}(x,{\hat{p}}_{x}),\;\;\;\;(y,P_{y})=\text{cumsum}(y,{\hat{p}}_{y}).$$

Then, the actual look-up tables representing true numerical cumulative distribution functions are defined as follows:

(13) (x^,P^x):x^=def[min⁡{𝒟x}​−​Δx2; x​+​Δx2],  P^x=def[0;Px],(y^,P^y):y^=def[min⁡{𝒟y}​−​Δy2; y​+​Δy2], P^y=def[0;Py],

where symbol [;] denotes vector concatenation.

The last operations make the numerical cumulative probability density function LUTs $\left(\hat{x},{\hat{P}}_{x}\right)$, of size *D_x_* + 1, and $\left(\hat{y},{\hat{P}}_{y}\right)$, of size *D_y_* + 1, available for the computation of the nonlinear model according to (4).

If the statistical model is monotonically increasing, then the look-up table $\left(\hat{x},{\hat{P}}_{x}\right)$ may be used as is. Otherwise, for monotonically decreasing models, it should be replaced by $\left(\hat{x},1-{\hat{P}}_{x}\right)$ in what follows. This being understood, we proceed by developing the last procedure steps for the monotonically-increasing-model case.

The nonlinear model is to be evaluated on an ordered set of *x*-points denoted here as *𝒳*. For consistency reason, it should hold *𝒳* ⊆ [min{*𝒟_x_*} max{*𝒟_x_*}]. Here, we propose the ordered set *𝒳* over which the model is to be evaluated to consist of *R* points equally spaced within the interval [min{*𝒟_x_*} max{*𝒟_x_*}], where *R* depends on the accuracy of the interpolation required for the model *f*(⋅). The quantity *R* appears as a finesse of partition for interpolation purpose. (Of course, the points where the model are evaluated do not need to be equally spaced if the application that the method is devised for needs a different choice.) The last step in the procedure is to numerically evaluate the quantity *P_x_*(⋅) over the set *𝒳*, then to evaluate the quantity *P_y_* ^−1^(⋅) over the values of function *P_x_*(⋅), namely,

(14) 𝒫x=definterp(x^,P^x,𝒳),  𝒴=definterp(P^y,y^,𝒫x).

Note that in the right-hand side of (14), the inverse function *P_y_* ^−1^(⋅) appears through the swapped LUT of function *P_y_*(⋅). The ordered sets *𝒳* and *𝒴*, both of size *R*, give rise to the look-up table representation (*𝒳*, *𝒴*) for the nonlinear model *f*(⋅) to be designed.

Two comments on the developed procedure for statistical modeling are in order.

- A macroscopic advantage of the procedure devised on here is that it does not involve any computation except for table handling (which may be efficiently implemented by procedures of sorting/searching on lists of real-valued numbers) and few simple multiplies/divisions and additions/subtractions on real-valued numbers.
- From the viewpoint of computer implementation, the operations on the data sets *𝒟_x_* and *𝒟_y_* may be effected in a parallel way until the very last step represented by operations (14).

A possible MATLABs-based implementation of the devised procedure is reported in Appendix A.

### 3.4. Computational complexity issues

Complexity is generally an important issue for neural systems: complex models may have overfitted parameters (they will model random and noisy components), whereas less-complex models may be underfitted (they will miss some key information). Neural systems are usually tuned to the data by controlling their complexity at the same time. A simple example is the selection of the number of hidden neurons in a multilayer feed-forward neural network architecture. The complexity, in this case, is expressed by the number of weights in between formal neural units.

In the present case, the complexity of the model (only one neural unit, but with LUT transfer function) depends upon the dimension of the LUT, which is determined in (7). In the present paper, thus, the complexity of the model is not subject to any learning or optimization process but it is provided a priori. The choice of rules (7) may thus be discussed with a view to computational complexity.

- The bin number should be a function of the available samples.
- It is difficult to envisage any universal a priori relationship between the number of bins and the number of available data samples. The rule (7) was selected empirically on the basis of a trial-and-error procedure on various data sets with different sizes (see Section 4).
- Depending on model complexity, it is to be expected that the model performances may degrade.
- As a thought on this topic, it might be advisable to optimize the model complexity on the basis of a measure of “smoothness” of the learnt model, based, for example, on its first- and second-order derivatives [37].
- Care should be taken about model complexity selection. In fact, any data-based model complexity selection procedure needs extra calculations and will inherently make model complexity higher.

**Figure 3:**
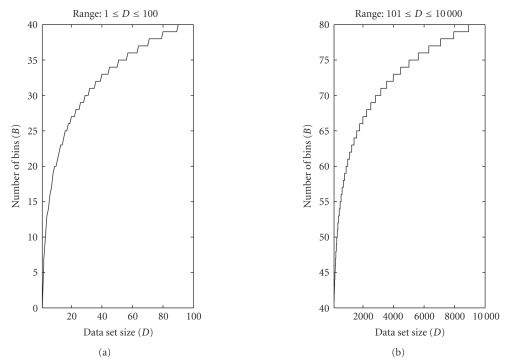
Model complexity versus the cardinality of a data set corresponding to rule (9).

A depiction of model complexity corresponding to rule (7) versus the cardinality of a data set is given in Figure 3. As it is readily seen, when the data set size is low the number of partition bins increases rapidly in order to follow the increasing complexity of data. Conversely, when the number of data samples is large, the model complexity increases slowly thus keeping limited. These observations were used as a rationale for the choice (7).

**Figure 4:**
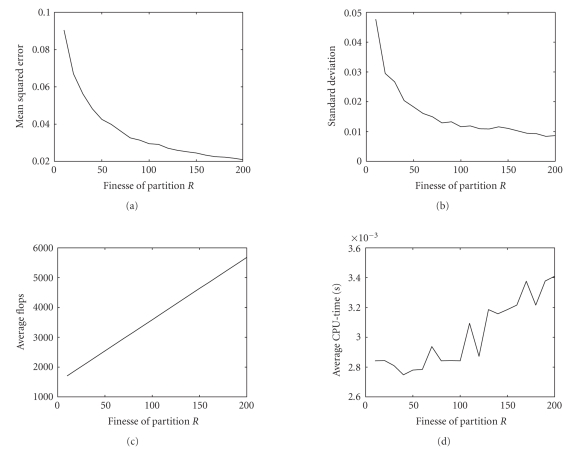
Mean-squared error and standard deviation (a)–(b) and average flops and CPU-time (c)–(d) versus finesse of partition for interpolation purpose on a test problem. (Averages are computed over 500 independent trials.)

We further tested the discussed algorithm on a simple modeling problem where the closed-form solution is known analytically (namely, a variant of the problem discussed with details in Section 4.1.2). The number of bins in the estimate of the required cumulative density functions was selected according to rule (7). The finesse of partition *R* was let to vary between 10 and 200 with step 10. The discrepancy between the estimated model and the actual model was measured as a mean-squared error. Also, the total number of flops and the CPU time required to run the whole modeling procedure was recorded over 500 independent trials on randomly generated data sets. In the experiment, the data sets were of different size, namely, with *D_x_* = 2354 samples and *D_y_* = 2544 samples. The obtained results were gathered in Figure 4. The results tell that the quality of approximation increases with the finesse of partition and the computational burden of the algorithm increases with the finesse of partition *R* as well. Perhaps, the most interesting result emerging from this experiment is that the number of flops as well as the CPU effort required by the algorithm to run on the used platform and within the used development environment are quite low (some 10^3^ flops versus some milliseconds). This makes the discussed procedure attractive from a computational viewpoint.

## 4. NUMERICAL EXPERIMENTS WITH SYNTHETIC AND REAL-WORLD DATA SETS

In this section, we apply the numerical procedure of Section 3.3 to the following.

- A synthetic data set with density distributions known in analytic form.
- A synthetic data set related to digital images for pattern recognition purpose.
- Sweet cherry blossom blooming data.
- Statistical characterization of the mechanical properties of polypropylene composites reinforced with natural fibers.
- Blood measurements data for trend discovery in laboratory analysis.

The algorithm consists of the set of equations discussed in Section 3.3 to be applied in the same order as they appear (see also the code on Appendix A).

### 4.1. Experiments on synthetic data

In the present section, we consider synthetic data sets whose density distributions are known in analytic form. In this case, the underlying model can even be computed by the algorithm proposed in [16] whose results may be compared with. We also consider synthetic data sets related to pattern recognition on black and white images.

#### 4.1.1. Synthetic data sets with known density distributions

Data within sets *𝒟_x_* and *𝒟_y_* were generated according to the following probability densities:

(15) $$\begin{array}{ll} p_{x}\left(x\right) & =\frac{\sec{\text{h}}^{\lambda -1}\left(x-\mu \right)-\lambda \;\sec{\text{h}}^{\lambda +1}\left(x-\mu \right)\sinh^{2}\left(x-\mu \right)}{\sqrt{2\pi \sigma }}\\ & \times \;\exp\left[-\frac{{\left(\sinh\left(x\right)\sec{\text{h}}^{\lambda }\left(x\right)-\sinh\left(\mu \right)\;\sec{\text{h}}^{\lambda }\left(\mu \right)\right)}^{2}}{2\sigma^{2}}\right],\\ p_{y}\left(y\right) & =\frac{1}{2}\left[\frac{1}{\sqrt{2\pi \sigma_{1}}}\;\exp\left(-\frac{{\left(y-\mu_{1}\right)}^{2}}{2\sigma_{1}^{2}}\right)+\frac{1}{\sqrt{2\pi \sigma_{2}}}\exp\left(-\frac{{\left(y-\mu_{2}\right)}^{2}}{2\sigma_{2}^{2}}\right)\right]. \end{array}$$

**Figure 5:**
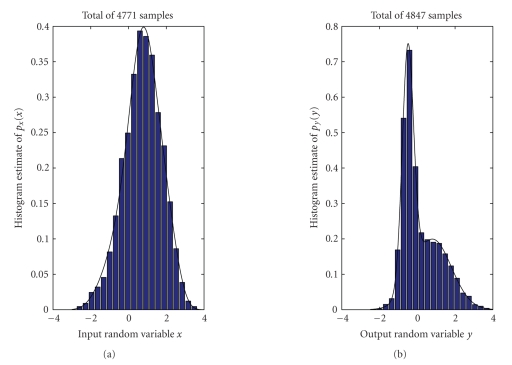
Probability density functions of the data sets *𝒟_x_* and *𝒟_y_*. (Experiment on synthetic data sets with known density distributions.) Note that in this example *D_x_* ≠ *D_y_*.

The details^3^ on the distributions as well as the algorithm used to generate the sample sets were given in [16]. The selected parameters values were *σ* = 1, *μ* = 0.8, *λ* = 0.5, *σ*_1_ = 0.3, *μ*_1_ = −0.5, *σ*_2_ = 1, and *μ*_2_= 0.8. The generated *x*-samples range in [−3 4] while the generated *y*-samples range in [−5/2 4]. The generated data distribute as shown in Figure 5. The data sets *𝒟_x_* and *𝒟_y_* were generated independently of each other and the set sizes *D_x_* = 4771 and *D_y_* = 4847 differ in this experiment.

**Figure 6:**
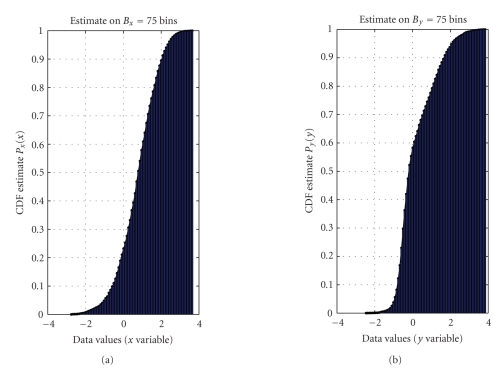
Cumulative distribution functions of the data sets *𝒟_x_* and *𝒟_y_* required by the modeling procedure. (Experiment on synthetic data sets with known density distributions.)

**Figure 7:**
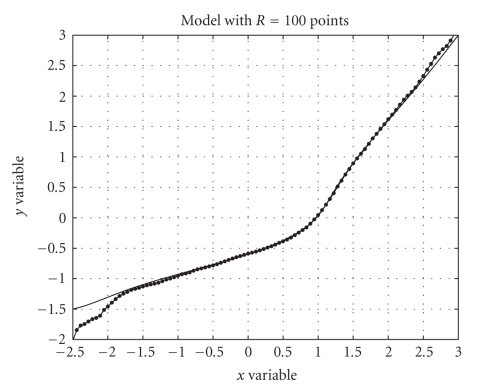
Model computed by the algorithm of Section 3.3 (solid dotted line) and exact nonlinearity computed with the algorithm presented in [16] (solid line). (Experiment on synthetic data sets with known density distributions.)

The cumulative distribution functions required by the modeling procedure are depicted in Figure 6 and the obtained model is depicted in Figure 7 along with the nonlinear function *f*(⋅) computed with the algorithm presented in [16]. As it can be readily appreciated, the numerical model obtained with the LUT-based algorithm of Section 3.3 is in excellent agreement to the nonlinear function computed on the basis of the closed-form probability density functions *p_x_*(⋅) and *p_y_*(⋅), except that near the “borders” of the variables domains.

#### 4.1.2. Synthetic data sets of black and white images

Shape analysis within digital images is a popular topic in pattern recognition. In order to fit the present statistical-modeling setting to shape analysis, let us suppose a black and white image is available and the data sets *𝒟_x_* and *𝒟_y_* are formed by pooling the (*x*, *y*) coordinates of the black dots within the image.

Here we can make use of a straightforward deduction from the closed-form expression of the nonlinear model *f*(⋅) give by (4): if the *x*-data and *y*-data distribute approximately in the same way (namely, *p_x_*(⋅) ≈ *p_y_*(⋅)) and the model is selected to be monotonically increasing, then the resulting model may only be of the kind *f*(*x*) ≈ *x*, that is, the model *f*(⋅) should closely resemble a straight line with unitary slope.

**Figure 8:**
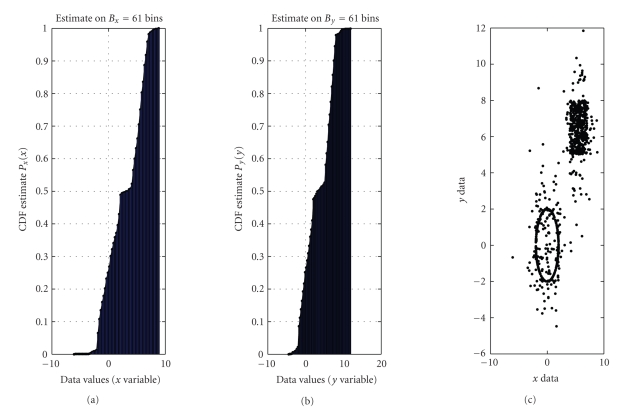
Rightmost panel: digital black and white image with an open circle and a filled-in square included. The image is corrupted by white Gaussian random noise. (Graphics might warp the shape of the image elements.) Cumulative distribution functions estimates are shown on the leftmost/central panels.

**Figure 9:**
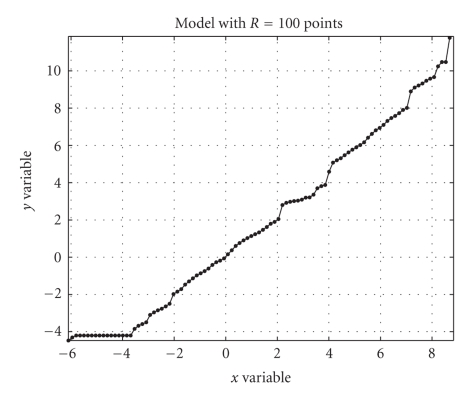
Model computed by the algorithm of Section 3.3 (Experiment on synthetic data sets of black and white images.)

In the context of shape analysis, the case that *x*-data and *y*-data distribute approximately in the same way might denote the presence of circles or squares within the image, as in the example depicted in Figure 8. The image is corrupted by 20% white Gaussian random noise with variances of the coordinates equal to (1,4) and with null correlation among the two noise components. The open circle is centered in (0,0) and has radius equal to 2. The filled-in square's left-bottom corner locates in (4,5) and its diagonal is of length $3\sqrt{2}$. The obtained numerical model is depicted in Figure 9. As it is readily seen, the model exhibits two approximately straight segments with unitary slope in the intervals [−2 2] and [4 7] as expected, interleaved by curly segments that are due to noise.

### 4.2. Experiments on “cherry blossom” data

Cherry blossom (*sakura*) has been celebrated for many centuries and occupies a quite prominent position in Japanese culture, being a well-known and ubiquitous symbol of Japan. Sakura are represented on all manner of consumer goods, including kimono, stationery, and dishware: cherry blossoms are an enduring metaphor for the ephemeral nature of life and, as such, are frequently depicted in art.

**Figure 10:**
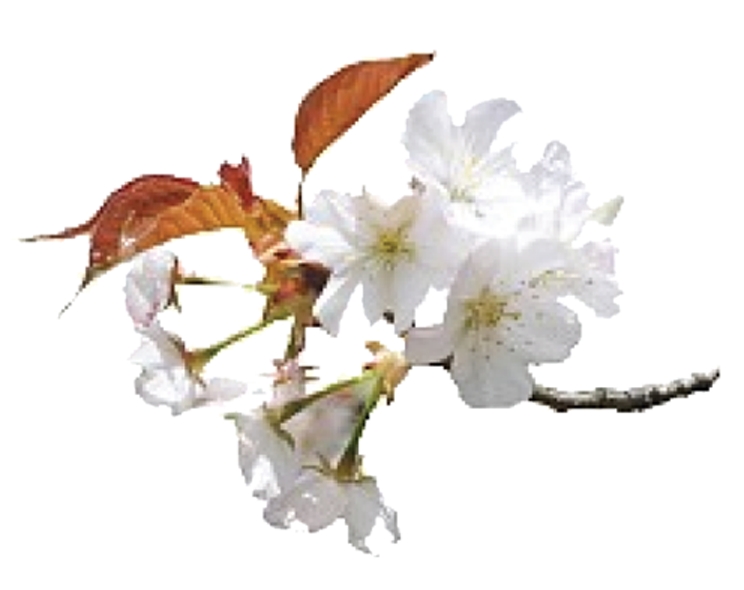
A Somei Yoshino cherry blossom.

There exist many dozens of different cherry-tree varieties in Japan, most of which bloom for just a couple of days during spring. Japan's most beloved variety of cherry blossom is the *Somei Yoshino* (see Figure 10). Its flowers are nearly pure white, tinged with the palest pink, especially near the stem. The flowers bloom, and usually fall within a week, before the leaves come out. Therefore, the trees look nearly white from top to bottom. Other categories of cherry blossom include *Yamazakura*, *Yaezakura*, and *Shidarezakura*.

Japanese people are used to celebrate the time of the year when cherry blossoms bloom with cherry blossom viewing parties (*hanami*) under the blooming trees. Most Japanese schools and public buildings have sakura trees in their outskirts. Since the fiscal and school year both begin in April, in many parts of Japan, the first day of work or school would coincide with the cherry blossom season.

**Figure 11:**
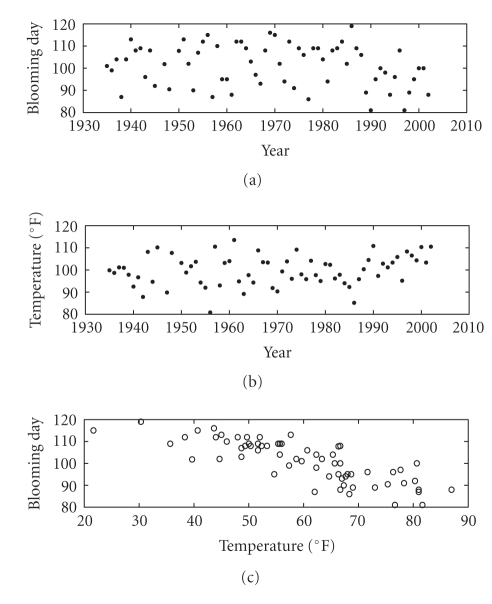
Cherry blossom recordings. (a)–(b) Blooming day since January 1st and conventional temperature (in Fahrenheit degrees) (1935–2002). (c) Data scatter plot.

The available data in the present experiment are the day when the cherry blossom bloom since January 1st within a cherry tree field and a conventional temperature (in °F) measured that day. The recordings span the years 1935–2002. The data are shown in Figure 11. As it may be readily seen (and intuitively understood), the lower the conventional temperature is, the later the cherry trees bloom. Therefore, if we identify the *𝒟_x_* data set with the set of blooming days since January 1st a year and the *𝒟_y_* data set with the set of conventional temperature measured during the blooming days a year, the model is expected to be monotonically decreasing.

**Figure 12:**
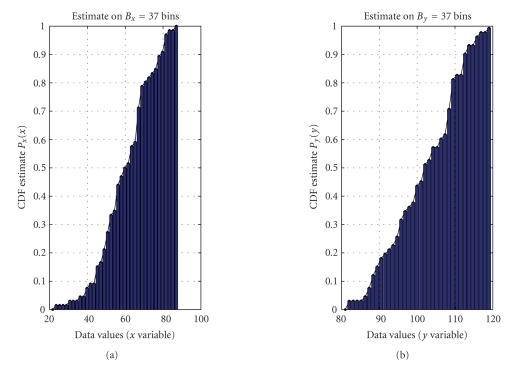
Cumulative distribution functions of the data sets *𝒟_x_* and *𝒟_y_* required by the modeling procedure. (Experiment on cherry blossom data.)

**Figure 13:**
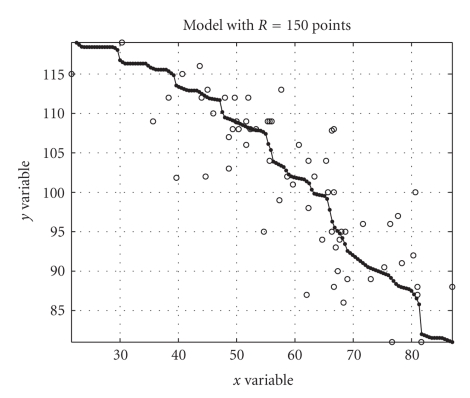
Model computed by the algorithm of Section 3.3 (Experiment on cherry blossom data. Dotted solid line: computed model. Circles: data points.)

The cumulative distribution functions required by the modeling procedure are depicted in Figure 12 and the obtained model is depicted in Figure 13. The computed model fits the data in a satisfactory way.

### 4.3. Reinforced composites mechanical behavior modeling: motivations and problem formulation

Synthetic fibers have been extensively used over the last years as reinforcement of plastic (polymeric) matrices. Fibers are incorporated into plastics with the aim of improving the mechanical properties of the polymer. The interest in the use of natural vegetal fibers as reinforcement has kept growing mainly due to their renewable origin, low price, and favorable environmental impact (natural fibers are biodegradable and natural-fiber-reinforced composites can be easily recycled). Therefore, ligno-cellulosic natural fibers represent an interesting alternative as substitutes for traditional synthetic fibers in composite materials.

On the other hand, in contrast with synthetic fibers, whose properties can be easily and univocally determined, natural fibers are characterized by a large dispersion of their characteristics. As a matter of fact, the behavior and the properties of natural fibers depend on many factors such as harvest period, weather variability, quality of soil, and climate of the specific geographic location. The variability of the properties of natural fibers is so high that it can also be observed among fibers belonging to the same plant. Such variability makes it difficult to predict the mechanical features of the yield composite and make it necessary to employ a statistical approach to model them.

In order to describe the characteristics of natural-fiber-reinforced composites, in [17] the use of statistical description of the quantities of interest was proposed. In particular, the following scheme was adopted.

**Figure 14:**
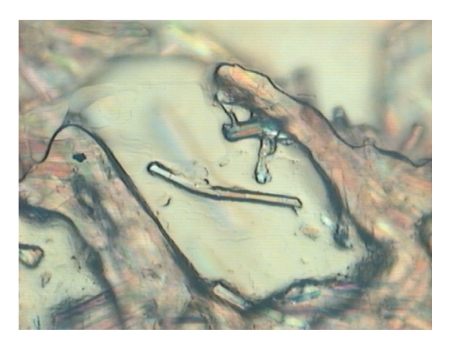
A shot of a matrix composite with flax fibers well visible embedded in. (Experiment on polypropylene composite data.)

(1) *Marginal statistical characterization of free fibers' geometrical/mechanical features*: the geometrical and mechanical features of interest of the natural fibers used to reinforce the composite may be characterized before production. In particular, the diameter (*d*) of any single fiber as well as the Young elasticity modulus (tensile strength, *E*) and the resistance (strain strength, *S*) are measured. The sets of these values are statistically characterized, namely, the probability density functions *p_d_*(⋅), *p_E_*(⋅), and *p_S_*(⋅) are estimated, in order to provide a statistical description of the geometrical/mechanical features of these fibers.

(2) *Adoption of a free-fibers mechanical model*: the mentioned geometrical/mechanical quantities, referred to the same set of fibers, are not independent. For instance, it is easily predictable that the elasticity of a free fiber depends on its diameter. In order to correlate these quantities, a popular model found in the literature is usually adopted. In particular, the Griffith model *E*(*d*) = *g*_1_ + (*g*_2_/*d*) is often employed because of its simple structure and because it embodies the fundamental intuition that the elasticity of a fiber is inversely proportional to its diameter. In [17], the Griffith's parameters *g*_1_ and *g*_2_ were computed by fitting the model to the available measures on the basis of a standard least-square procedure.

(3) *Composite production and in-slab fibers measurement*: the measured fibers were then employed to yield a reinforced composite matrix. The production process breaks and warps the fibers within the composite: their mechanical features change but still explain the mechanical properties of the yield composite. In order to characterize these properties it is therefore necessary to examine the geometry of the fibers within the composite (which appears as a transparent matter after fusion and leaves the fibers visible, see, e.g., Figure 14): the length and diameter of the residual fibers within the composites are measured. It is important to note that, at this stage, just the geometric features of the residual fibers may be actually measured via visual inspection, while the mechanical properties of the residual fibers cannot be measured directly. Such mechanical properties are therefore to be predicted through the use of the fitted Griffith model applied to the diameter values of the fibers within the composite. As explained later, this choice results to be only partially sensible.

(4) *Modeling of composite's mechanical properties*: the statistical characterization of the mechanical properties of the composite slab may now be predicted trough the Halpin-Tsai model [17] which, on the basis of the modal value of the free-fibers geometrical/mechanical features and of the set of length/diameter values of the residual fibers within the composites and other few production data, furnishes a series of mechanical properties values, namely, *E_c_* and *S_c_*, of the composites. This is true under the hypothesis that the composite features may be computed as the weighted distribution of the mechanical values pertaining to homogeneous fibers. Namely, the Halpin-Tsai model, in conjunction to a proper PDF-estimation technique, is used to estimate the quantities

(16) $$\begin{array}{c} p_{E_{c}}(E_{c})=\int_{{𝒟}_{c}}\int_{{ℒ}_{c}}p_{E_{c}\;|\;d_{c},l_{c}}(E_{c}|\delta ,\ell )p_{d_{c},l_{c}}(\delta ,\ell ),d\delta \;d\ell ,\\ p_{S_{c}}(S_{c})=\int_{{𝒟}_{c}}\int_{{ℒ}_{c}}p_{S_{c}\;|\;d_{c},l_{c}}(S_{c}|\delta ,\ell )p_{d_{c},l_{c}}(\delta ,\ell ),d\delta \;d\ell , \end{array}$$

where *p_E__c_*(⋅) and *p_S__c_*(⋅) denote the distributions of the two mechanical properties of the composite, *p_E__c_*|*d_c_*,*l_c_*(⋅ | ⋅, ⋅) and *p_S__c_*|*d_c_*, *l_c_*(⋅ | ⋅, ⋅) denote the effect of embedded fibers geometry on composite's mechanical characteristics, and *p_d__c_*, *l_c_*(⋅, ⋅) denotes the weighting geometric factor. The intervals *𝒟_c_* and *ℒ_c_* denote the value ranges of the diameters and lengths of the fibers within the composite.

(5) *Marginal statistical characterization of composite's mechanical features*: a statistical characterization of the distribution of the values *E_c_* and *S_c_* returns the modal values of these quantities as a final result. These predicted values may be finally compared to the measured values, after mechanical tensile/strain characterization of the composite slab, in order to validate the quality of the prediction. Such comparison showed that the results obtained by using this procedure are more accurate than the results obtained without passing trough the statistical characterization of the single-fiber measures. However, analysis also showed that the Griffith model, computed on the basis of the geometrical features of the free fibers and employed in order to predict the value of the mechanical features of the fibers embedded in the polypropylene composite on the basis of their geometrical features, is not completely reliable.

The unreliability of the Griffith model in the present application may be explained by the fact that it was originally thought to for synthetic (glass) fibers and does not take into account the different mechanical behavior of the original fibers during composite production. Also, for large diameter values, the Griffith model may loose physical meaning because it may return negative values for the tensile strength. We may consider replacing the two-parameter Griffith model with the LUT-based neural model of Section 3. The motivations of this replacement are the following.

- As the neural model has free (monotonic) shape, no preknowledge that might bias the solution is required nor intentionally embedded into the resulting model, except for the monotonicity of the model. It is worth noting that, according to basic mechanical considerations and to Griffith model itself, the relationship *E* = *f*(*d*) should be monotonically decreasing.
- The standard least-squares fitting used to optimize the Griffith model (or any other parametric model) works under the hypothesis that the measured values of *E* and *d* are available as coupled, namely, in the form (*d_k_*, *E_k_*), with *k* ranging between 1 and the total number of available measures. This might not, however, be the case: it is well possible that during the measurement session a nonnegligible fraction of free fibers cannot be fully characterized in diameter, tensile and strain strength (in particular, the last feature is rather difficult to measure due to its inherently destructive character). Therefore, standard fitting-based modeling does not appear to be appropriate here.
- Further about the measurement difficulties, the measures obtained in this kind of characterization are always somewhat unreliable, for instance, because of the tendency of the fibers to form agglomerates and to fray, which make it difficult to obtain accurate measurements of the their geometrical features. Statistical characterization inherently provides a way to weight and interpret correctly the available results.

In the present contribution, we focus on the tensile strength prediction of reinforced composites, thus we focus primarily on modeling the relationship *E* = *f*(*d*) for the free fibers. As mentioned, a set of measures of the diameter values and a set of fibers' elasticity modulus values are available for a bunch of free fibers.

**Figure 15:**
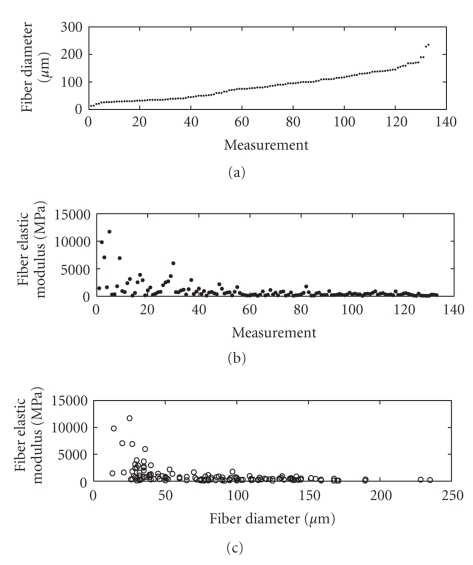
Polypropylene composite recordings. (a)–(b) Diameters(*μ*m) and elastic modulus (MPa). (c) Data scatter plot.

**Figure 16:**
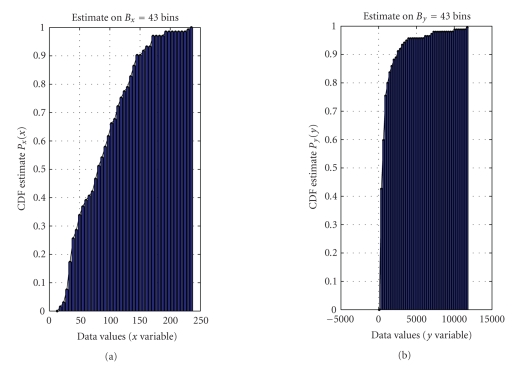
Cumulative distribution functions of the data sets *𝒟_x_* and *𝒟_y_* required by the modeling procedure. (Experiment on cherry blossom data.)

**Figure 17:**
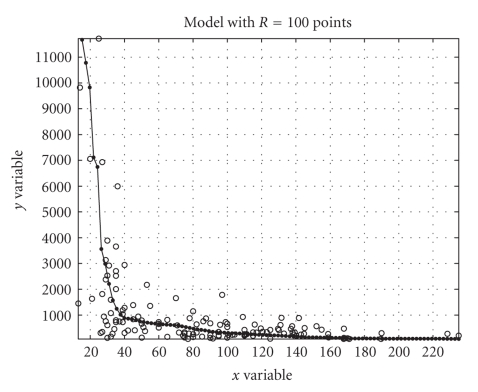
Model computed by the algorithm of Section 3.3 (Experiment on polypropylene composite data.)

After the statistical distributions *p_d_*(⋅) and *p_E_*(⋅) are estimated, we aim at finding a model *E* = *f*(*d*) that transforms the probability density of the diameters into the probability density of the elastic moduli. Our basic hypothesis is that the same model also explains the relationship between the diameters and moduli themselves. As mentioned, no prior knowledge is required for modeling. The available data in the present experiment are the measured diameters expressed in micrometers (*μ*m), which we identify with the *𝒟_x_* data set and elastic moduli expressed in Mega-Pascal (MPa), which we identify with the *𝒟_y_* data set. The data are shown in Figure 15. The estimated cumulative distribution functions required by the modeling procedure are depicted in Figure 16 and the obtained model is depicted in Figure 17. The computed model fits the data in quite a satisfactory way. Also, by comparing the model obtained with the proposed algorithm with the model obtained in the previous work [17], it can be concluded that the new model looks to be more adherent to the actual measurements.

### 4.4. Blood measurements data sets

The 1982 annual meeting of the American Statistical Association (ASA) was held during August in Cincinnati (OH, USA). At that meeting, the ASA Committee on Statistical Graphics sponsored an “exposition of statistical graphics technology.” The purpose of this activity was to more fully inform the ASA members about the capabilities of computer graphics in statistical work.

**Figure 18:**
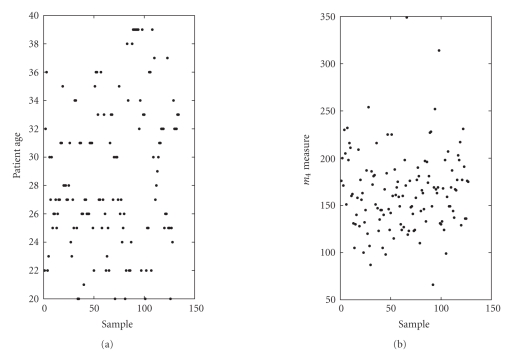
Blood measurements data sets: data set *𝒟_x_* is the pooled set of patients ages (years), data set *𝒟_y_* is the pooled set of *m*_4_ measures (unknown measure unit). The *m*_4_ data set contains missing measures, therefore the sizes of the data sets *𝒟_x_* and *𝒟_y_* differ.

As a common database that the statistical analysis methods were to be tested on, a set of biomedical data was made available, which arose in a study to develop screening methods to identify carriers of a rare genetic disorder. Four measurements *m*_1_, *m*_2_, *m*_3_, and *m*_4_ on blood samples were made available.^4^ Because the disease is rare, there are only a few carriers of the disease from whom data are available. The data came in two files, one for carriers and one for noncarriers of the disease. The data were stripped off of the patients names and other identifiers, otherwise the data were made available as received by the analyst [45]. Each vendor or provider of statistical graphics software participating in the exposition analyzed these data using their software and prepared tabular, graphical, and text output illustrating the use of graphics in such analysis and summarizing their conclusions: the purpose of the analysis was to develop a screening procedure to detect carriers and to describe its effectiveness. As additional information to data themselves, the following warning was provided: “experts in the field have noted that young people tend to have higher measurements.” We take as data set *𝒟_x_* the pooled set of patients ages (expressed in years) and as data set *𝒟_y_* the pooled set of *m*_4_ measures (unknown measure unit). The *m*_4_ measures contain “holes” therefore the sizes of the data sets *𝒟_x_* and *𝒟_y_* differ. The data are shown in Figure 18.

**Figure 19:**
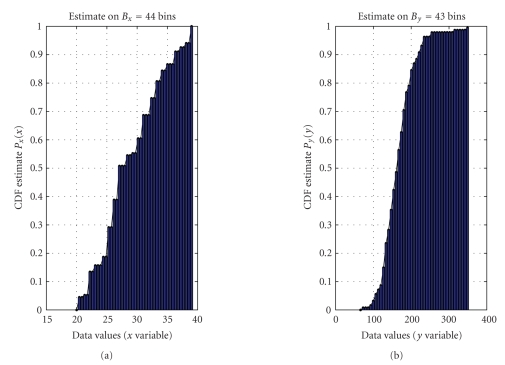
Cumulative distribution functions of the data sets *𝒟_x_* and *𝒟_y_* required by the modeling procedure. (Experiment on blood measurements data.)

**Figure 20:**
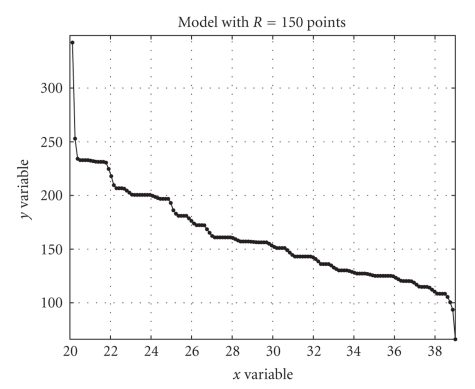
Model computed by the algorithm of Section 3.3 (Experiment on blood measurements data.)

The purpose of the modeling effort proposed here is to quantify the trend of *m*_4_ measure versus patient's age, under the hypothesis that such dependency is decreasing, as suggested by analysts. The estimated cumulative distribution functions required by the modeling procedure are depicted in Figure 19 and the obtained model is depicted in Figure 20. The result of modeling seems to evidence a quasilinear trend in the decrease of the *m*_4_ value versus age, in a range [22 38].

## 5. CONCLUSION

In the present paper, we discussed the problem of bivariate statistical modeling via neural (nonlinear) systems that are able to match their input-output statistic to the statistic of data sets, a relationship among which is sought for.

We proceeded under the missing-at-random assumption for the incomplete data and considered bivariate statistical modeling via a method that looks partially resembling to a regression method. Statistical modeling was given here an interpretation as pooled-statistic matching problem for a nonlinear system. Namely, instead of considering the *x* ∈ *𝒟_x_* and *y* ∈ *𝒟_y_* variables as paired and to look for a nonlinear model that best fits the variables values, we consider only cumulative information that arise by pooling the values within the data sets *𝒟_x_* and *𝒟_y_*. The neural system nonlinear transference thus matches the probability distributions of the variables, rather than the variables' values themselves. As a consequence, the proposed statistical modeling technique allows to cope with the modeling problem when the size of the two data sets does not match and/or when the pairing relationship of the values within *x* ∈ *𝒟_x_* and *y* ∈ *𝒟_y_* is unknown. Also, as the individual data in the sets *𝒟_x_* and *𝒟_y_* are not accessed directly by the modeling procedure, if the data sets of the phenomena under modeling are not available but only their pooled cumulative statistical distributions are available, the modeling process may take place anyway. The resulting nonlinear model fits the data in a probability-density-function transformation sense.

A key point of the method is that the quantities of interest as well as the designed model were proposed to be represented in terms of paired lists of real numbers termed LUTs, which were proven to provide an efficient way of representing and handling the variables appearing within the devised statistical modeling algorithm. A prominent advantage of the procedure is the lack of hard computational requirement except for LUT handling (which consists in sorting/searching on lists of numbers) and few simple algebraic operations on numbers. It was underlined that an inherent restriction of the method is that the developed theory requires the model to be monotonic. The model shape is otherwise unrestricted, being thus free of any other shape constraint and resulting free of biasing effects inherently tied to other modeling methods.

In order to assess the numerical statistical modeling method proposed in the present work, numerical experiments performed on synthetic as well as real-world data sets, both of matching/unmatching sizes were conducted. The proposed algorithm was applied to modeling a synthetic data set with density distributions known in analytic form and a synthetic data set related to digital images for pattern recognition purpose. Also, it was applied to the modeling of sweet cherry blossom blooming data, to the statistical characterization of the mechanical properties of polypropylene composites reinforced with natural fibers as well as to the modeling of blood measurements data for trend discovery in laboratory analysis. The results of numerical experiments showed that the computed models fit the data in a satisfactory way and result otherwise sensible and reasonable.

Two technical aspects of the discussed method may raise some concerns, namely, the following.

- *Intrinsic discretization of the input/output function when look-up tables are used*: while the use of LUTs makes the handling of the algorithm easy, the intrinsic discretization causes errors with respect to the continuous case. In particular, the interpolation as a way to obtain better solutions might rise some concerns about the accuracy of function representation. The present Author has investigated in previous paper the effect of changes in bin size/numbers, for example, in the context of estimating the entropy of a random variable whose samples only are available [46]. This problem is also discussed in the book [47].
- *Scalability for high-dimensional data*: it would be interesting considering the extension of the discussed bivariate statistical modeling procedure to multivariate statistical modeling, namely, to the case that a many-to-one variables model is needed of. Such extension will need a theoretical basis to be developed as well as a set of convenient numerical representation/handling procedures to be devised subsequently.

We are currently seeking for an extension of the discussed isotonic bivariate statistical modeling approach to a many-to-one modeling procedure. The main challenge here is how to formulate the multivariate statistical modeling by statistic matching. A possible solution under investigation seems to involve partial differential equations on the modeling function based on joint probability density functions of the “independent” variables and of the “dependent” variable. From the technical/implementation side, the extension of traditional (2-variable) LUTs to multivariable LUTs looks straightforward.

## Figures and Tables

**Figure 21:**
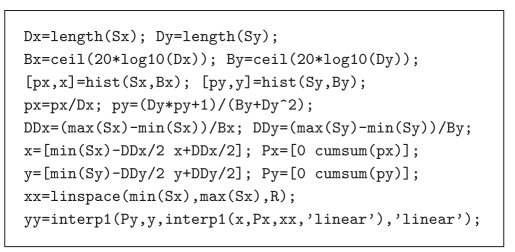
MATLAB-based implementation of the (monotonically increasing) modeling procedure described in Section 3.3

## Footnotes

^1^Longitudinal study of American youth (LSAY), started in 1987 as an ongoing four-year panel study of middle and high school science and mathematics education. The base-year sample consisted of 2,829 10th grade students from 51 randomly selected public schools across the United States. Survey instruments were completed by the sampled students, their teachers and parents. Achievement tests that focused on mathematics and science knowledge and higher-order thinking skills were administered in the fall of 1987, 1988, and 1989. The LSAY has then kept going on and is now located at Michigan State University. The LSAY database is the longest longitudinal study of students and young adults ever conducted in the United States. Currently, all LSAY students are being surveyed to obtain updated information on their educational and occupational attainment. The 2007 survey will look at social, political, and community participation.

^2^The definitions of cumulative density functions are given here for probability density functions defined on the whole real line. Such definitions hold even for probability density functions defined over intervals, with the trick that the density functions are attributed a zero value outside of their supports.

^3^The hyperbolic secant function is defined as $\text{sech}(x)\overset{\text{def}}{=}2/(e^{x}+e^{-x})$, while the hyperbolic sinus function is defined as $\sinh(x)\overset{\text{def}}{=}(e^{x}-e^{-x})/2$, with *x* ∈ ℝ.

^4^The whole data set was gathered from the StatLib website http://lib.stat.cmu.edu

## APPENDIX

### A. IMPLEMENTATION OF THE MODELING PROCEDURE

In the present appendix, we report a MATLAB-based implementation of the modeling procedure described in Section 3.3 In the code reported in Figure 21, the data set *𝒟_x_* is represented by the vector Sx while the data set *𝒟_y_* is represented by the vector Sy. The learnt model is represented by the couple of vectors xx and yy. The operators hist, cumsum, and iterp1 are proper MATLAB primitives.

We underline that the code reported in Figure 21 is not an iterative learning procedure: the execution of the program one time produces the sought-for model.
